# The mechanism of perioperative intravenous lidocaine in regulating the inflammatory response: A review

**DOI:** 10.1097/MD.0000000000039574

**Published:** 2024-09-06

**Authors:** Jingyi Wang, Qifan Bian, Xiaoqing Chen, Yue Feng, Lantian Zhang, Peng Chen

**Affiliations:** a Department of Anesthesiology, China-Japan Union Hospital of Jilin University, Changchun, China.

**Keywords:** anti-inflammatory, inflammatory response, intravenous infusion, lidocaine, pain

## Abstract

Perioperative inflammatory responses are a series of endogenous immune responses produced by the body following surgical trauma. Excessive inflammatory response weakens the body’s ability to repair surgical trauma and reduces the body’s defense against the invasion of harmful factors, leading to a series of complications, such as infections, pain, and organ damage, which prolong the length of hospitalization and increase the risk of death. Lidocaine is a classical local anesthetic widely used in clinical practice because of its local anesthetic and antiarrhythmic effects. Several recent studies have shown that lidocaine modulates the body’s inflammatory response, and that its anti-inflammatory properties can lead to analgesia, organ protection, and improved postoperative recovery. In this paper, we introduce the mechanism of the modulating effect of lidocaine on the perioperative inflammatory response and its clinical application, to provide a reference for the clinical prevention and treatment of the perioperative inflammatory response.

## 1. Introduction

The perioperative inflammatory response is a series of endogenous immune responses produced by the body in the face of tissue damage caused by surgery.^[1]^ A moderate inflammatory response helps the body resist inflammatory trauma; however, an excessive inflammatory response weakens the body’s ability to repair surgical trauma and reduces the body’s defense against harmful factors, leading to a series of postoperative complications and thus slowing down the process of postoperative recovery. Lidocaine is a classic local anesthetic drug that has been widely used in clinical practice owing to its good local anesthetic and antiarrhythmic effects. Recent studies have shown that perioperative intravenous lidocaine plays a positive role in modulating the inflammatory response,^[2]^ however, the mechanism of this action and its clinical efficacy are still unclear. This review aims to summarize the literature and provide a basis for the use of lidocaine in the prevention and clinical management of perioperative inflammatory responses.

## 2. Overview of the concept and mechanisms of perioperative inflammatory response

The perioperative inflammatory response is a series of endogenous immune responses to surgery-induced tissue damage.^[1]^ Tissue ischemia and reperfusion injury, blood transfusion, neurally-mediated inflammatory responses, and mechanical ventilation can further induce and exacerbate inflammatory responses. Current findings generally suggest that the mechanism of the perioperative inflammatory response involves the release of “alarms,” that is, endogenous factor damage-associated molecular patterns (DAMPs), from injuries such as surgical trauma.^[3]^ DAMPs activate immune cells, including neutrophils and monocytes, through DAMP receptors on the cell surface, releasing inflammatory mediators, and inducing a series of immune processes.^[4]^ The strength of the perioperative inflammatory response determines the outcome and prognosis of surgical treatment to some extent. A moderate inflammatory response is beneficial for assisting the body in defending itself against injurious external stimuli. However, excessive inflammation weakens the body’s ability to repair surgical trauma, reduces the body’s defense against harmful factors, and leads to a series of complications, such as infection, pain, pulmonary complications, renal injury, and postoperative delirium, thus slowing down the recovery process and causing unnecessary medical burden. Therefore, in clinical practice, there is a need to actively take measures to intervene in the perioperative inflammatory response to reduce or abate the inflammatory response and improve the patient’s prognosis. Currently, perioperative inflammatory responses are usually combated by intervening in surgical procedures, such as reducing surgically induced tissue trauma and shortening the duration of surgery, or by adjusting the anesthetic strategy and the use of anti-inflammatory drugs.^[5]^ It is now known that anesthetic drugs such as isoproterenol and isoflurane may reduce perioperative inflammatory factor expression, hydroxyethyl starch may stabilize the glycocalyx of cells after inflammation induction, and regional anesthetics have great potential to modulate neuroinflammation.^[5,6]^ Recent studies have shown that perioperative intravenous lidocaine can positively modulate the inflammatory response, providing new ideas for perioperative inflammation management.^[2]^

## 3. Pharmacological properties of lidocaine

Lidocaine was first synthesized by Lofgren in 1935 and was officially approved for use in 1948. Lidocaine is a classic local anesthetic of the amide class, and has been widely used in clinical practice for its local anesthetic and antiarrhythmic effects. When used for local anesthesia, it blocks sodium channels in the nerve cell membrane and, therefore, the generation and conduction of action potentials. In addition to the classical local anesthetic effects, an increasing number of studies have shown that intravenous infusion of lidocaine has many potential effects, such as anti-inflammatory, organ-protective, and anticancer effects.^[7–9]^ Its anti-inflammatory properties in the perioperative period are currently attracting a lot of attention, and the analgesic effect from its anti-inflammatory properties has been demonstrated.^[10]^ The potential benefits of intravenous lidocaine may persist long after the end of infusion and long after the drug is metabolized to a non-biologically active state. As typical perioperative blood levels block only a very small percentage of sodium channels, the role of intravenous lidocaine in achieving anti-inflammatory properties may be derived primarily from other mechanisms.^[7]^

## 4. Mechanisms by which lidocaine modulates the inflammatory response

The potential anti-inflammatory mechanism of lidocaine may be due to its ability to block polymorphonuclear neutrophil (PMN) initiation. PMN initiation occurs when a cell is exposed to certain mediators and subsequently activated by a second mediator, resulting in an overreaction that releases cytokines and reactive oxygen species, leading to endothelial cell, vascular, and even organ damage. It has been suggested that lidocaine may reduce neutrophil adhesion and inhibit the release of superoxide anion, as well as blocking the release of inflammatory mediators such as interleukins (IL), interferon-gamma, high-mobility group protein B1, tumor necrosis factor-alpha (TNF-α) and transforming growth factor-beta.^[11,12]^ Kolle et al^[13]^ also found that lidocaine inhibited the migratory capacity and extent of PMN migration. A study by Lin et al^[14]^ found that lidocaine could exert anti-inflammatory effects by inhibiting the hypoxia-inducible factor-1α mediated glycolytic pathway in rats and significantly inhibiting the secretion of lipopolysaccharide-mediated inflammatory factors in macrophages, suggesting a potential mechanism for the anti-inflammatory effects of lidocaine.

Studies suggest that lidocaine may produce anti-inflammatory effects by affecting cell signaling. Mitogen activated protein kinases (MAPKs) are key signal transduction factors for the conversion of extracellular signals into intracellular responses in eukaryotic cells, and extracellular regulated protein kinases is a major member of the MAPK pathway family, whereas nuclear factor-kappa B is a protein with transcriptional activation functions, and a study showed that lidocaine suppresses inflammatory responses by inhibiting activation of the MAPK/extracellular regulated protein kinases/nuclear factor-kappa B pathway.^[15]^ Ma et al^[16]^ used hypoxia/re-oxidation-induced type II alveolar epithelial cells to simulate ischemia/reperfusion in vitro and found that the hypoxia/reoxygenation-induced increase in p38MAPK-related proteins in A549 cells was significantly downregulated by lidocaine treatment, and that the levels of IL-6, IL-8, and TNF-α were significantly elevated in lidocaine-treated cells as compared to controls. This evidence suggests that the modulation of the MAPK signaling pathway may be an effective target for the inhibitory effect of lidocaine on the inflammatory response. Li et al^[17]^ demonstrated that lidocaine may alleviate neuropathic pain and neuroinflammation and inhibit the inflammatory response by down-regulating the high-mobility group protein B1 inhibitory macrophage inflammatory protein-1 alpha/chemoline receptor1/chemoline receptor5 signaling pathway. MicroRNAs (miRNAs) are important regulators of biological processes such as apoptosis. It has been shown that miRNA may have a key role in regulating inflammatory responses, and inflammation may dysregulate miRNA expression, while at the same time dysregulation of miRNA expression may be accompanied by increased activation of inflammatory factors. In the study of Rancan et al^[18]^ cells in the lidocaine-intervention group showed decreased levels of expressed miRNA, as well as decreased levels of the inflammatory markers TNF-α and IL-1. This indicates that the protective effect on the inflammatory response in the group given lidocaine intravenously may be mediated by altering miRNA expression. Suppressors of cytokine signaling (SOCS) are a family of 8 proteins, of which SOCS3 is a negative regulator that plays a key role in signaling processes induced by extracellular signals in the inflammatory response. Zheng et al^[19]^ showed that intrathecal injection of lidocaine increased the pain threshold in experimental mice, and upregulated SOCS3 to inhibit microglial cell activation, thereby suppressing inflammation and reducing neuropathic pain. Zhang et al also concluded that lidocaine upregulated SOCS3 to inhibit the inflammatory response. Lidocaine can also inhibit neuroinflammatory responses by upregulating SOCS3 levels through an AMPK-dependent signaling pathway.^[20]^

Lidocaine can modulate inflammatory responses by interfering with the receptors in the central nervous system. Glutamate receptors are important excitatory amino acid receptors within the central nervous system, and are divided into 2 categories: ionotropic and metabotropic receptors. Ionotropic receptors include N-methyl-D-aspartate receptors, kainic acid receptors, and a-amino-3-hydroxy-5-methyl-4-isoxazole propionic acid receptors, which are coupled to ion channels to mediate fast signaling. Metabotropic receptors are coupled to G proteins and produce slow physiological effects when activated. Chiu et al^[21]^ demonstrated that lidocaine pretreatment reduces kainic acid-induced activation of hippocampal microglia and gene expression of pro-inflammatory factors, such as IL-1b, IL-6, and TNF-α. While glycine receptors are important inhibitory neurotransmitter receptors in the central nervous system, they are also necessary co-stimulators of N-methyl-D-aspartate-type excitatory glutamate receptors. Some studies have indicated that low concentrations of lidocaine enhance glycine receptor function. Lidocaine or its metabolite, n-ethylglycine, may be responsible for the systemic effects by inhibiting cell activation through competitive inhibition of the glycine transporter protein glycine transporter protein 1.^[22]^

In conclusion, lidocaine may modulate the inflammatory response and attenuate the release of inflammatory mediators by inhibiting inflammatory cells, modulating signaling, and interfering with central neuroreceptor transmission. The above studies explain the possible targets of lidocaine in regulating the inflammatory response; however, further basic research and clinical trials are needed to explore the potential mechanisms and clinical effects of intravenous lidocaine in regulating the inflammatory response, and to provide a reference for possible ways of preventing and controlling the inflammatory response in the clinic, as well as for the direction of future research.

## 5. Clinical application

Many clinical studies have been conducted to explore the mechanism of perioperative intravenous lidocaine infusion in modulating inflammation, as well as its safety and clinical application. In recent years studies have shown that the analgesic effect of perioperative intravenous lidocaine is mainly achieved due to its anti-inflammatory effects. In most studies patients are usually first given an initial dose of 1.5 to 2 mg/kg of lidocaine, which is infused at 2 to 4 mg/kg/h during anesthesia to achieve a blood concentration of 1 to 3 mg/mL^11^. The clinical use and outcomes of perioperative intravenous lidocaine infusion in some major types of surgery are reviewed below, to provide a clinical reference.

### 5.1. Abdominal surgery

Many studies have demonstrated the positive effects of perioperative intravenous lidocaine infusion in modulating the inflammatory response in patients undergoing abdominal surgery. Intravenous lidocaine infusion reduces postoperative pain and opioid use.^[23–25]^ Nakhli et al^[26]^ reported similar findings in renal surgery. In a study of patients undergoing laparoscopic bariatric surgery, Sun et al^[27]^ compared intravenous lidocaine infusion with ultrasound-guided transversus abdominis fascia block and found that patients receiving intravenous lidocaine had better postoperative analgesia. This may be due to the anti-inflammatory properties of lidocaine and the fact that systemic application of lidocaine can also directly block the sodium channels of nerve fibers transmitting pain and reduce the incidence of postoperative nociceptive hypersensitivity. In abdominal surgery, pain mainly originates from the somatic pain caused by skin incision, visceral pain caused by mechanical pulling, ischemia, the inflammatory response, and neuralgia caused by neuroinflammatory release due to nerve injury. Therefore, abdominal analgesia is particularly suitable for multimodal analgesia, to compensate for the inadequacy of a single class of analgesic drugs. Studies have shown that intravenous lidocaine helps reduce the inflammatory response to abdominal surgery and improves the quality of postoperative recovery. Wang et al^[28]^ found that the use of lidocaine in the perioperative period improved postoperative cognitive dysfunction in patients with colorectal cancer, with lower white blood cell counts in the lidocaine group than in the control group on the first postoperative day, a lower incidence of cognitive dysfunction than in the control group on the third and seventh postoperative days, and significantly higher Mini-Mental State Examination scores than in the control group. This may be related to the neuroprotective effect of lidocaine by inhibiting the neuroinflammatory response. Peng et al^[29]^ found that intravenous infusion of lidocaine reduced the degree of short-term postoperative pain in patients undergoing hysteroscopy, in addition to reducing the need for intraoperative opioids and the incidence of postoperative sore throat. Notably, in a study by Xu et al,^[30]^ it was found that in patients undergoing laparoscopic hysterectomy, intravenous lidocaine combined with dexmedetomidine infusion was associated with lower levels of inflammatory factors and pain intraoperatively and 2 hours postoperatively compared to patients receiving lidocaine or dexmedetomidine alone, indicating the value of the combination of drugs. This evidence suggests that perioperative intravenous lidocaine has a positive effect in patients undergoing abdominal surgery, both intraoperatively and postoperatively.

### 5.2. Orthopedic surgery

Patients undergoing spinal surgery may have long term postoperative low back pain. Ibrahim et al^[31]^ found that the mean pain scores of patients receiving intravenous lidocaine were significantly lower than those of the control group at 48 hours, 3 months postoperatively, and at long-term postoperative follow up. Intravenous lidocaine infusion has been shown to have short- and long-term benefits in the postoperative period for patients undergoing spinal surgery and to improve their postoperative quality of life, suggesting that perioperative lidocaine infusion may be of value for use in patients undergoing major spinal surgery. The duration of action of lidocaine exceeds the infusion time and plasma half-life, suggesting that lidocaine may act by preventing hypersensitivity reactions in the central or peripheral nervous system, or both, and attenuate the postoperative adverse events due to the presence of a systemic inflammatory response that may occur in patients undergoing spinal surgery.

### 5.3. Breast surgery

The efficacy of the intravenous administration of lidocaine in breast surgery is controversial. In 2015, Couceiro et al^[32]^ administered 3 mg/kg of lidocaine intravenously to mastectomy patients within the first 24 hours of surgery, and there was no additional analgesic effect or reduction in opioid consumption compared to patients treated with placebo. However, a multicenter study in 2021 demonstrated that continuous intraoperative infusion of lidocaine had a positive impact on long-term postoperative analgesia in patients undergoing breast cancer surgery, and the safety of lidocaine application in breast cancer surgery was demonstrated in that study.^[33]^ This suggests that different doses and time points of administration may have different effects on clinical outcomes; however, the exact reasons for this are unclear, and further studies are needed.

### 5.4. Head and neck surgery

Intravenous lidocaine infusion during intracranial tumor surgery improved cerebral relaxation after dural opening and reduced intraoperative opioid consumption.^[34]^ Wang et al^[35]^ found that systemic lidocaine infusion improved the postoperative quality of recovery scores in patients undergoing upper airway surgery, reduced the intraoperative opioid dosage, lowered postoperative nausea and vomiting at 2 days postoperatively, and improved the quality of early postoperative recovery. However, in a study of ear, nose and throat surgery, Wallon et al^[36]^ found that intravenous lidocaine did not provide any additional analgesic benefit.

### 5.5. Pediatric surgery

In children between the ages of 18 months and 18 years undergoing laparoscopic appendicitis surgery, Kaszyński et al^[37]^ showed that an intravenous infusion of lidocaine reduced the need for intraoperative opioids. In colonoscopy, intravenous lidocaine infusion reduced visceral pain and propofol dosage in children.^[38]^ Intravenous lidocaine to reduce opioid use may reduce postoperative nausea and vomiting. Zouche et al^[39]^ compared the effects of lidocaine, dexamethasone, and placebo on postoperative vomiting and postoperative feeding in children undergoing tonsil surgery; intravenous lidocaine infusion was not advantageous in this study. Perioperative intravenous lidocaine infusion has been less well-studied in pediatric patients and is more difficult to evaluate in children than in adults; however, further studies are needed to clarify this.

### 5.6. Cardiac surgery

In a study on cardiac surgery, Klinger et al^[40]^ found that intravenous application of lidocaine reduced the transcerebral inflammatory response; however, Klinger et al^[41]^ found that intravenous lidocaine during and after cardiac surgery did not reduce cognitive decline 6 weeks postoperatively. The protective mechanism and benefits of intravenous lidocaine infusion on postoperative cognitive function are unclear. It has been indicated that lidocaine has the potential to improve microvascular perfusion over anti-inflammatory and antioxidant mechanisms, as it is associated with better tissue perfusion and oxygen delivery, potentially improving organ function and combating postoperative organ dysfunction.^[16,42]^ The above studies suggest the value of intravenous lidocaine infusion in perioperative organ protection through its anti-inflammatory effects; however, there are few relevant clinical trials, and further research is needed.

## 6. Summary and outlook

In summary, intravenous lidocaine infusion has a positive effect on the perioperative inflammatory response. Intravenous lidocaine may play a role in modulating the inflammatory response as well as having potential effects such as analgesia, organ protection, and improved postoperative recovery through a series of potential mechanisms; however, current clinical trials on intravenous lidocaine infusion in modulating the inflammatory response are not comprehensive. The dosage and time point of lidocaine application as well as its clinical effects need further research. The mechanism of intravenous lidocaine infusion in modulating the perioperative inflammatory response has not been clarified, although current basic studies have explained some potential targets of action. Further basic and clinical experiments are needed to explore and validate the mechanism of action, optimal dose, mode of administration, and application effects of intravenous lidocaine, and to provide further clues for studies related to perioperative inflammatory responses.

## Author contributions

**Conceptualization:** Jingyi Wang, Peng Chen.

**Data curation:** Jingyi Wang, Qifan Bian, Xiaoqing Chen, Yue Feng, Lantian Zhang.

**Writing – original draft:** Jingyi Wang, Qifan Bian, Xiaoqing Chen, Yue Feng, Lantian Zhang.

**Writing – review & editing:** Jingyi Wang, Peng Chen.
